# Absorption and metabolism of conjugated α-linolenic acid given as free fatty acids or triacylglycerols in rats

**DOI:** 10.1186/1743-7075-3-8

**Published:** 2006-01-20

**Authors:** Mélanie Plourde, Jean-Pierre Sergiel, Jean-Michel Chardigny, Stéphane Grégoire, Paul Angers, Jean-Louis Sébédio

**Affiliations:** 1 UMR INRA-ENESAD Flavic, 21065 Dijon, France; 2 Department of Food Science and Nutrition, INAF/Dairy Research Center (Stela), Université Laval, Sainte-Foy, Québec, G1K 7P4, Canada; 3 INRA-Université d'Auvergne, Laboratoire de Nutrition Humaine, 63 009 Clermont-Ferrand, France

## Abstract

**Background:**

Conjugated linoleic acid (CLA) is a group of polyunsaturated fatty acids which have been extensively studied in the past two decades. However, conjugated octadecatrienoic acid such as *cis*-9,*trans*-11,*cis*-15 and *cis*-9,*trans*-13,*cis*-15, recently identified, have not been extensively investigated. This work presents bioavailability and tissue incorporation of a mixture of conjugated octadecatrienoic (CLnA) acids ingested as free fatty acids (FFA) and triacylglycerols (TAG).

**Results:**

Male Wistar rats were fed rumenic acid (RA: *cis*-9,*trans*-11 18:2) and a CLnA mixture (*cis*-9,*trans*-11,*cis*-15 18:3 and *cis*-9,*trans*-13,*cis*-15 18:3) as FFA and TAG for 8 days. RA and CLnA were both totally absorbed when given as FFA as well as TAG. Both isomers of CLnA as FFA or TAG were incorporated into neutral lipids. Metabolites up to 22:6 conjugated isomers were present in liver and plasma phospholipids of rats fed the CLnA diets.

**Conclusion:**

Finally, CLnA are as well absorbed as RA *in vivo *and their incorporation into tissues and bioconversion are similar when ingested as FFA or as TAG.

## Background

Conjugated linoleic acid (CLA) is a group of polyunsaturated fatty acids (PUFA) found in ruminant meat (about 0.4% of the total lipids) and milk products (about 1% of the total lipids) with a conjugated double bonds system [1]. Among the CLA family, rumenic acid (RA), *cis*-9,*trans*-11 18:2 is the major conjugated isomer found in ruminant fat. Many health benefits have been attributed to RA isomers like inhibition of carcinogenesis in rats [2] whereas CLA mixtures have demonstrated abilities to modulate atherosclerotic plaque formation in rabbits and hamsters [3,4] and to decrease body fat mass in mice [5]. Although many investigations were done regarding prevention of diseases, few studies have investigated the impact of the ingested CLA form (FFA, TAG or FAME) on tissue metabolism and absorption [6]. In this respect, Fernie *et al. *[6] showed that CLA absorption in humans as FFA and TAG is similar as illustrated by their occurrence into chylomicrons, while the absorption was lower for fatty acid methyl esters.

**Figure 1:**
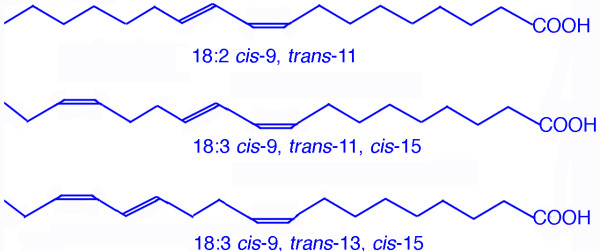
Structure of both isomers included in the CLnA mixture compared to rumenic acid.

Conjugated alpha linolenic acid (CLnA) is another group of PUFA. One isomer has recently been reported in milk fat at a level of about 0.03% of total fatty acids [7]. The *cis*-9,*trans*-11,*cis*-15 18:3 isomer results from the biohydrogenation of α-linolenic acid. The *cis*-9,*trans*-11,*cis*-15 18:3 isomer combines the conjugated double bond system of RA and the n-3 double bond of α-linolenic acid (Figure 1) which confers this fatty acid a high bioactive potential. The metabolic fate of a synthetic mixture of FFA of two conjugated α-linolenic acids containing equimolar concentrations of *cis*-9,*trans*-11,*cis*-15 18:3 and *cis*-9,*trans*-13, *cis*-15 18:3 isomers has already been studied in rats [8]. In that study CLnA isomers had been given intragastrically after a two weeks lipid deprivation period in order to enhance the bioconversion. [8]. Moreover, no investigations were done regarding the absorption efficiency.

The aim of the present study was to determine the absorption efficiency, incorporation and metabolism of a mixture of conjugated α-linolenic acid in growing rats under nutritional and physiological conditions.

## Results

### Animals

RA intake ranged from 330 ± 30 mg/day for the TAG group to 338 ± 14 mg/day (means ± SD) for the FFA group. CLnA intake ranged from 323 ± 25 mg/day for the FFA group to 334 ± 20 mg/day for the TAG group. No significant differences on the total diet consumption were observed among the four groups.

At the end of the feeding period, the weights of the animals were not significantly different, 227 ± 6 (means ± SD); 221 ± 10 in groups fed RA and CLnAs as FFA and 218 ± 7; 223 ± 6 in groups fed the RA and the CLnAs as TAG.

### Effects of dietary fat on fatty acid digestibility and absorption

Table 2 shows the apparent digestibility efficiency for total fatty acids and for each fatty acid. Digestibility of total fatty acid was not significantly different between the four experimental groups. Each group had a lower absorption of saturated fatty acids compared to monounsaturated and polyunsaturated fatty acids. RA and CLnA have been totally absorbed as FFA and TAG suggesting that under these conditions, there is no difference in absorption.

**Table 1:** Fatty acid composition (%) of the lipid fraction of each diet

	**FFA**		**TAG**	
	
**Fatty acids**	**RA**	**CLnA**	**RA**	**CLnA**
**16:0**	3.4	3.4	4.0	4.2
**16:1 *c*-9**	0.1	0.1	0.1	0.1
**18:0**	3.0	3.0	2.9	2.9
**18:1 *c*-9**	68.7	67.5	69.1	67.8
**18:2 *c*-9,*c*-12**	9.9	9.8	10.0	10.0
**18:3 *c*-9,*c*-12,*c*-15**	1.2	1.1	1.2	1.1
**18:2 *c*-9,*t*-11**	10.5	0.3	10.0	0.2
**18:2 *t*-10,*c*-12**	ND	0.5	ND	0.5
**18:3 *c*-9,*t*-11,*c*-15 and *c*-9,*t*-13,*c*-15**	ND	11.1	ND	10.7
**Others**	3.2	3.7	2.7	3.0

**Table 2:** Apparent digestion efficiency (%) of the dietary fatty acids in Wistar rats fed RA- or CLnA-supplemented diets as free fatty acids (FFA) or as triacyglycerols (TAG)

	**FFA**				**TAG**			
	
	**RA**		**CLnA**		**RA**		**CLnA**	
	
	Mean	SD	Mean	SD	Mean	SD	Mean	SD
**Total fatty acids**	98.0	0.3	98.2	0.2	98.2	0.2	98.0	0.3
**Fatty acids**								
**16:0**	89.6	0.8	89.6	0.5	86.6a	1.1	90.0	0.8
**18:0**	83.1	2.0	85.8	2.0	80.2	1.7	80.4	2.4
**18:1 *c*-9**	98.6	0.3	99.2	0.2	99.2	0.2	98.9	0.3
**18:2 *c*-9,*c*-12**	99.4	1.0	99.3	0.1	99.5	< 0.1	99.4	0.08
**18:3 *c*-9,*c*-12,*c*-15**	100.0	< 0.1	100.0	< 0.1	100.0	< 0.1	100.0	< 0.1
**18:2 *c*-9, *t*-11**	99.9	< 0.1	100.0	< 0.1	99.9	< 0.1	100.0	< 0.1
**18:2 *t*-10,*c*-12**	ND	< 0.1	100,0	< 0.1	ND	ND	100.0	< 0.1
**18:3 *c*-9,*t*-11,*c*-15 and *c*-9,*t*-13,*c*-15**	ND	< 0.1	100,0	< 0.1	ND	ND	99.9	< 0.1

Values with different letters are significantly different (P < 0,05) and compare the fatty acid (RA and CLnA) under the same form (FFA or TAG)

Values with an * are significantly different (P < 0,05) and compare the form (FFA and TAG) within one diet (RA and CLnA).

### Effects of dietary fat on the fatty acid profile in liver, plasma and adipose tissue

The main fatty acids in liver, plasma and adipose are presented in Table 3. RA and CLnA accumulated preferentially in neutral lipids. No major differences between RA and CLnA accumulation were seen whatever the dietary form, FFA or TAG. RA and CLnA accounted for up to 3% of the total FAME of the adipose tissue. On the other hand, slight differences were found in monounsaturated fatty acids of the liver neutral lipids between rats fed the FFA form of RA and CLnA. The rats fed the TAG form had significant differences (P < 0.05) of saturated fatty acids profile of plasma PL. The animals fed CLnA as FFA had lower content of arachidonic acid in liver and plasma PL.

**Table 3:** Fatty acid composition (%) of liver, plasma and adipose tissue of Wistar rats fed RA or CLnA as FFA or TAG. Results are expressed as means (*SD*) of 6 determinations.

				**Saturated**		**Monounsaturated**		**Polyunsaturated**		**Conjugated**	
**Lipid form**	**Organ**	**Lipid class**	**Diet**	**C16:0**	**C18:0**	**16:1 n-7**	**18:1 n-9**	**18:2 n-6**	**20:4 n-6**	**RA**	**CLnA**

	Liver	PL	RA	19.14	23.21	1.19^a^*	9.25	6.67^a^*	23.08^a^	0.23	ND
			CLnA	18.65	22.85*	1.91^b^	9.17*	8.68^b^	20.20^b^	ND	0.42
		NL	RA	30.67	3.14	4.46^a^*	48.41a*	2.66	0.86	1.46	ND
			CLnA	31.73	3.33	6.56^b^	42.63	3.17	0.77	ND	2.18
	
FFA	Plasma	PL	RA	27.24	25.15	0.79^a^*	12.24^a^	14.20	10.25^a^	0.24	ND
			CLnA	27.59	25.73	1.19^b^	10.93^b^	15.17	8.79^b^	ND	0.25
		TAG	RA	25.71	2.13	3.69^a^*	53.98^a^*	4.64^a^	0.30	1.39	ND
			CLnA	0.69	1.86	5.90^b^	46.76^b^	5.86^b^	0.30	ND	2.69
		CE	RA	26.70	0.71	3.15^a^*	16.83	15.68^a^	43.73	0.11	ND
			CLnA	9.98*	0.66*	4.93^b^	15.53*	17.53^b^	41.50	ND	0.32
	
	Adipose	TAG	RA	22.91	2.35^a^	6.56*	49.98	6.79	0.26	3.44	ND
			CLnA	23.93*	1.95	6.80	47.79	7.16*	0.14	ND	3.79

	Liver	PL	RA	18.68^a^	22.59^a^	1.97	8.93a	7.9	21.18	0.29	ND
			CLnA	17.20^b^	24.15^b^	1.84	7.75	8.1	21.45	ND	0.32
		NL	RA	33.93	2.89	7.04	42.89	2.58	0.71	1.24	ND
			CLnA	34.81	3.04	7.59	40.49	2.54	0.59	ND	1.79
	
TAG	Plasma	PL	RA	28.51^a^	24.61^a^	1.35	11.29^a^	13.48	9.75	0.26	ND
			CLnA	25.95^b^	26.79^b^	1.29	10.12^b^	14.35	10.24	ND	0.27
		TAG	RA	27.80	1.80	5.97	46.37	5.61	0.34	2.63	ND
			CLnA	27.09	1.88	6.60	45.32	5.76	0.34	ND	3.01
		CE	RA	8.75	0.66	5.01	14.83^a^	15.34	44.73	0.26	ND
			CLnA	8.72	0.55	5.45	12.74	16.77	44.98	ND	0.47
	
	Adipose	TAG	RA	25.72	2.24^a^	8.12	46.69	6.05	0.17	3.03	ND
			CLnA	26.68	2.00	7.87	45.14	6.30	0.15	ND	3.27

Values with different letters are significantly different (P < 0,05) and compare the fatty acid (RA and CLnA) under the same form (FFA or TAG)

Values with an * are significantly different (P < 0,05) and compare the form (FFA and TAG) within one diet (RA or CLnA)

### CLnA Metabolites

CLnA metabolites are shown in Table 4. The *cis*-9,*trans*-11,*cis*-15 18:3 was elongated and desaturated up to *cis*-4,*cis*-7,*cis*-10,*cis*-13,*trans*-15,*cis*-19 22:6 while the *cis*-9,*trans*-13,*cis*-15 18:3 was metabolized up to *cis*-5,*cis*-8,*cis*-11,*trans*-15,*cis*-17 20:5. Conjugated metabolites accumulated in both neutral lipids and polar lipids. However, the *cis*-7,*cis*-10,*cis*-13,*trans*-15,*cis*-19 22:5 and the *cis*-4,*cis*-7,*cis*-10,*cis*-13,*trans*-15,*cis*-19 22:6 were exclusively detected in liver and plasma phospholipids. No differences in the amount of the metabolites were found between the two dietary forms given (FFA or TAG). This suggests that there was no difference in the metabolic fate (elongation and desaturation) of CLnA when ingested as FFA and as TAG.

**Table 4:** Metabolites of RA and CLnA (%) found in different organs of the Wistar rats fed RA and CLnA as FFA and TAG.

		**Organ**				
		
		**Plasma**		**Liver**		**Adipose**
		
**Dietary form**	**Fatty acids**	**PL**	**TAG**	**PL**	**NL**	**TAG**
**FFA**	20:3 *c*-11,*t*-13,*c*-17	0.058	0.101	0.154	0.136	0.092
	20:4 *c*-8,*c*-11,*t*-13,*c*-17	0.010	0.088	0.039	0.092	0.062
	20:5 *c*-5,*c*-8,*c*-11,*t*-13, *c*-17	0.013	0.022	0.031	0.019	0.003
	*20:5 *c*-5,*c*-8,*c*-11,t-15, c-17	0.009	0.012	0.021	0.010	0.015
	22:5 *c*-7,*c*-10,*c*-13,*t*-15, *c*-19	0.016	ND	0.049	ND	ND
	22:6 *c*-4,*c*-7,*c*-10,*c*-13,*t*-15,*c*-19	0.015	ND	0.062	ND	ND

**TAG**	20:3 *c*-11,*t*-13,*c*-17	0.053	0.113	0.136	0.100	0.078
	20:4 *c*-8,*c*-11,*t*-13,*c*-17	0.012	0.104	0.038	0.068	0.056
	20:5 *c*-5,*c*-8,*c*-11,*t*-13, *c*-17	0.015	0.028	0.028	0.014	0.004
	*20:5 *c*-5,*c*-8,*c*-11,t-15, c-17	0.010	0.013	0.022	0.009	0.014
	22:5 *c*-7,*c*-10,*c*-13,*t*-15, *c*-19	0.017	ND	0.047	ND	ND
	22:6 *c*-4,*c*-7,*c*-10,*c*-13,*t*-15,*c*-19	0.021	ND	0.068	ND	ND

ND, not detected

*Metabolite of *c*-9,*t*-13,*c*-15 18:3 FA, all other metabolite are from *c*-9,*t*-11,*c*-15 18:3

FA

## Discussion

Most of the studies so far published on CLA focussed on their putative effects on biological function and prevention of metabolic disorders. However, few studies considered the effect of the dietary form (FFA or TAG) on their bioavailabilty and metabolism [6], while dietary supplement are often available as free fatty acids. The major reason may be that TAG are more expensive. On the other hand, only one study has reported that force-fed conjugated α-linolenic acids (CLnA) given as FFA were incorporated into liver and adipose tissue of PUFA deprived rat and reported that both CLnA isomers were following the elongation/desaturation pathway [8]. Otherwise, no studies have shown their absorption efficiency in nutritional conditions.

In the present study, the experimental fatty acids (RA and CLnA mixture) were given as FFA or as randomized TAG and were equally incorporated into tissues (Table 2). No significant differences in absorption levels were found between those two lipid forms. This suggests that the dietary form did not modify the incorporation of RA and of CLnA into tissues under our experimental conditions. These data are in agreement with the study of De Schrijver *et al. *[10] who compared the absorption of fish oil fatty acids given as FFA and TAG. While in the present study RA and CLnA were totally absorbed, a previous study using lymphatic recovery showed that a CLA mixture given as FFA was less absorbed than linoleic acid [11]. No study was so far published on the lymphatic recovery of CLnA. However, it has already been shown that RA absorption as TAG did not behave like a common fatty acid because of its conjugated structure or its *trans *double bound in Δ^11 ^position or both [13]. In the present study, we also demonstrate that CLnA mixture is preferably incorporated into neutral lipids similar to RA. The incorporation of *trans *fatty acids in NL has long time been demonstrated with elaidic acid (*trans*-9 18:1) and shows that it follows the metabolic pathway of saturated fatty acids [14]. Moreover, it has been hypothesized by Martin *et al. *[15] that linoleic acid, generally in high concentration in diets, may act as a competitor against RA for its incorporation into phospholipids and for the enzyme needed for the elongation/desaturation pathway. This hypothesis was also sustained by Banni *et al. *[16]. The Δ^6 ^desaturase is the limiting enzyme for the bioconversion of linoleic acid to arachidonic acid [17] and is also needed by n-3 fatty acids in the elongation/desaturation pathway. CLnA competes as well for this enzyme as seen by the lower arachidonic acid content in liver PL in rats fed CLnA. Metabolites from both CLnA isomers were found in NL as in PL. The *cis*-9,*trans*-13,*cis*-15 18:3 isomer was elongated and desaturated up to 20:5 n-3 conjugated isomer while *cis*-9,*trans*-11,*cis*-15 18:3 was elongated and desaturated up to 22:6 n-3 conjugated isomer. This end product of the elongation/desaturation pathway was obtained without essential fatty acid deprivation before the experimental period and with a short term nutritional intake. This shows that the *cis*-9,*trans*-11,*cis*-15 18:3 can compete for enzyme with essential fatty acids such as α-linolenic acid to follow the elongation/desaturation pathway. The conjugated isomers of 22:6 n-3 and 22:5 n-3 were found exclusively in polar lipids of liver and plasma while the RA metabolites were found in neutral lipids as demonstrated by Sebedio *et al. *[18]. This shows that conjugated metabolites of *cis*-9,*trans*-11,*cis*-15 18:3 could interfere in the fatty acid composition of membranes which affect their properties such as fluidity and permeability. Other studies are needed to confirm this hypothesis.

## Conclusion

This study demonstrated that an equimolar mixture of two CLnA isomers, *cis*-9,*trans*-11,*cis*-15 18:3 and *cis*-9,*trans*-13,*cis*-15 18:3 has the same apparent absorption efficiency than RA when ingested under nutritional and physiological conditions. Both CLnA isomers are mainly incorporated into neutral lipids. They are metabolized by the elongation/desaturation pathway similar to α-linolenic acid with the *cis*-9,*trans*-11,*cis*-15 18:3 isomer being converted into conjugated 22:6 n-3 and with the *cis*-9,*trans*-13,*cis*-15 18:3 isomer being converted into conjugated 20:5 n-3. The biological impact of the ingestion of these fatty acids has to be considered as they could interfere with lipid metabolism.

## Methods

### Fatty acids

Free fatty acids of conjugated linoleic acid (CLA) and conjugated linolenic acid (CLnA) were gratefully provided by Naturia Inc. (Sherbrooke, Canada). The high oleic sunflower oil and the linseed oil were purchased from Lesieur (Asnières, France) and Robbe (Compiègne, France), respectively.

### Triacylglycerol (TAG) synthesis

All solvents were purified by distillation before utilization. TAG of CLA and CLnA were synthesized as described by Kodali *et al. *[19]. TAG were then purified on a silica column using hexane/diethyl ether (80:20 v/v) as elution solvent. Thin-layer chromatography on silica gel using hexane/diethyl ether (80:20 V/V) as elution solvent showed that the purified fraction consisted entirely of TAG.

### Animals

Six week-old males (n = 24) Wistar rats were purchased from Elevage Janvier (Le Genest Saint Isle, France). The initial weight of the animals was 82 ± 7 g. (mean +/- SD). The rats were individually housed in stainless steel cages and were placed in an animal house at constant temperature (22 ± 1°C) and relative humidity (55–60%) with a 12-h light-dark cycle according to French regulation (authorization A21200 for the animal house and 21CAE056 for one of the author). They were adapted during 4 days with a semi-liquid diet (see below) before being allocated to one of the 4 dietary groups (see below). Six animals were allocated to each group. The average starting weight in each group was 101 ± 1 g. At the end of the 8 days experimental period, the animals were fasted for 16 hours and weighted. They were then anesthetised with isofurane and exsanguinated by abdominal aortic blood puncture. The collected heparinized blood extraction was centrifuged at 700 g, and +4°C during 10 min. The plasma was then collected and stored at -20°C until lipid extraction and analysis. The liver and a sample of the epididymal adipose tissues were also excised and stored in a chloroform/methanol (2:1 v/v) solution at -20°C until lipid extraction.

### Diets

The experimental diets were fed in a semi-liquid form in order to facilitate the determination of food consumption. The diets contained (g/kg) wheat starch: 460, sucrose: 220, casein: 180, high oleic sunflower/linseed oils mixture 98:2 w/w: 50, mineral mixture: 50, cellulose: 20, CLA or CLnA: 10, vitamins mix: 10. The diet used for the adaptation of the rats contained 60 g/kg of a high oleic sunflower/linseed oil mixture 98:2 w/w instead of 50 g/kg. CLA and CLnA were studied as FFA and TAG. The fatty acid content of each diet is summarized in Table 1. Food was exchanged every two days. At that time, the remaining semi-liquid food was removed and weighed to determine food intake. The animals were weighed twice per times a week. The faeces were collected each day, pooled and frozen at -20°C.

### Faeces lipid extraction and analysis

Before being lyophilized, the total faeces collected from each rat were frozen at -80°C for 24 h. The dried faeces were cleared of impurities, weighted and crushed with a ball crusher. A sample of 1 g of the homogenized powder was used for lipid extraction using a mixture of chloroform/methanol/formic acid (2:1:0.02 v/v/v) as a Folch modified procedure [20,21]. The extract was then evaporated to dryness and left for one night under vacuum in a dessicator. The total lipid extract was then weighted and dissolved in chloroform in gauge flask for conservation at -20°C. The apparent digestion efficiency (ADE) was calculated as follow [22].



The quantification of each fatty acid in the faeces was done by GC analysis of their methyl esters using an internal standard (17:0). Thus, the total fecal lipids were saponified under mild conditions before methylation. Briefly, an aliquot of 2 mL of the fecal extract in chloroform in gauge flask (around 4 mg/mL) was evaporated under nitrogen flow and 5 mL of a KOH/ ethanol (95%) 1 M solution was added to the dry extract. The solutions were left in the dark at room temperature for 16 h. After addition of 5 mL of acetic acid 1 N and 5 mL of water, the free fatty acids were extracted with 3 × 5 mL hexane/diethyl ether 1:1 v/v. The solution was centrifuged at 700 g for 3 minutes. The total extract in hexane was then evaporated and methylated using BF_3 _in methanol (14%) at room temperature for 20 min. The resulting FAME were then analysed by GC using a Hewlett-Packard serie II gas chromatograph equipped with a BPX70 capillary column (SGE, Melbourne, Australia, 120 m × 0.25 mm i.d×0.25 μm film thickness) with an FID detector and a *split less *injector. The oven temperature program was: 60°C for 1 min; 20°C/min up to 170°C for 60 min; 5°C/min up to 210°C for 15 min. FAME were identified using authentic standards and quantitative data were obtained using the Diamir software (JMBS Developments, Le Fontanil, France).

### Liver, plasma and adipose tissues lipids

Liver and adipose tissue lipids were extracted using a mixture of chloroform/methanol (2:1 v/v) as described by Folch *et al. *[20]. Liver lipids were separated into phospholipids and neutral lipids as described by Juanéda and Rocquelin [23]. Plasma lipids were extracted according to Moilanen and Nikkari [24] and separated into PL, CE and TAG on silica gels plates with hexane/diethyl ether/acetic acid (80/20/1 V/V/V) as elution solvent. All lipid classes were methylated with sodium methoxide in methanol (2N). The resulting FAME were then analysed by gas-liquid chromatography as described above for fecal fatty acids. FAME were identified using authentic standards. RA metabolites were identified as described by Sébédio *et al. *[18] and CLnAs metabolites were identified as described by Destaillats *et al. *[8]. Qualitative data were obtained using the Diamir software (JMBS Developments, Le Fontanil, France).

### Statistical analysis

Data are presented as mean ± SD and comparison were done by using SAS software as a one way FA (CLA or CLnA) or lipid form (FFA or TAG) ANOVA procedure. P values of less than 0.05 were considered as significant.

## Competing interests

The author(s) declare that they have no competing interests.

## Authors' contributions

MP carried out the experimental study, a part of the lipid analysis and drafted the manuscript, JPS participated in the design and the coordination of the study and carried out the experimental study, JMC participated in the design and the conceive of the study and helped to draft the manuscript, SG carried out lipid analysis, PA participated in the design of the study and helped to drafted the manuscript, JLS participated in the design, the conceive and the coordination of the study and helped to draft the manuscript.
